# Selective Electrochemical Detection of SARS-CoV-2 Using Deep Learning

**DOI:** 10.3390/v14091930

**Published:** 2022-08-30

**Authors:** Ozhan Gecgel, Ashwin Ramanujam, Gerardine G. Botte

**Affiliations:** Chemical and Electrochemical Technology and Innovation (CETI) Laboratory, Department of Chemical Engineering, Texas Tech University, Lubbock, TX 79409, USA

**Keywords:** COVID-19 diagnosis, COVID deep learning, electrochemical biosensor, electrochemical SARS-CoV-2 detection, differential diagnosis

## Abstract

COVID-19 has been in the headlines for the past two years. Diagnosing this infection with minimal false rates is still an issue even with the advent of multiple rapid antigen tests. Enormous data are being collected every day that could provide insight into reducing the false diagnosis. Machine learning (ML) and deep learning (DL) could be the way forward to process these data and reduce the false diagnosis rates. In this study, ML and DL approaches have been applied to the data set collected using an ultra-fast COVID-19 diagnostic sensor (UFC-19). The ability of ML and DL to specifically detect SARS-CoV-2 signals against SARS-CoV, MERS-CoV, Human CoV, and Influenza was investigated. UFC-19 is an electrochemical sensor that was used to test these virus samples and the obtained current response dataset was used to diagnose SARS-CoV-2 using different algorithms. Our results indicate that the convolution neural networks algorithm could diagnose SARS-CoV-2 samples with a sensitivity of 96.15%, specificity of 98.17%, and accuracy of 97.20%. Combining this DL model with the existing UFC-19 could selectively identify SARS-CoV-2 presence within two minutes.

## 1. Introduction

Early diagnosis of infectious diseases could be crucial for saving lives and restricting the disease spread. Often, health care professionals record enormous data to diagnose a disease. These large amounts of data have been accumulated over the years and has become challenging for professionals to process them in real-time. Machine learning (ML) and deep learning (DL) algorithms were developed for processing large amounts of data in a relatively short period of time and predict the results. As a result, artificial intelligence (AI) techniques have been used as a support in making clinical decisions [1]. These algorithms have gained immense recognition since 2016 when the Food and Drug Administration started approving DL- and AI-based devices for multiple specialties [2].

Hitherto, ML and AI have been used for diagnosing various infectious diseases. Multiple strategies ranging from supervised to unsupervised learning have been adopted for diagnosing diseases such as dengue [3], hepatitis B [4], tuberculous meningitis [5], etc. The accuracy of predicting the disease with the best models range anywhere between 79% and 98% [3,4,5]. Results from these literature strongly signify the impact ML has on diagnosing infectious diseases.

More recently, the advent of the COVID-19 pandemic has attracted the use of ML and DL for early and rapid diagnosis of the disease [6,7,8]. Several researchers around the world have resorted to multiple approaches for this purpose. They range from training algorithms with X-ray and CT images [7,9,10], spectral features [4], clinical and lab data [11], blood parameters [12], and other clinical features [13]. Although machine learning can classify COVID-19-affected individuals by these methods, the data collection techniques suffer from large storage space requirements, invasive sampling, long durations, and high costs.

Moreover, these studies focus on patients affected only with COVID-19. Specifically diagnosing COVID-19 in patients with flu, cold, or pneumonia is complicated [7,14]. The focus must be shifted towards differentially diagnosing COVID-19 in patients potentially with other infections. Studies in the past have shown that COVID-like symptoms could be misleading and conceal the possibility of any underlying disease [15]. As a result, it is highly possible for an erroneous diagnosis especially owing to some shared features among COVID-19 and other pneumonias [16]. This underlines the need for alternate diagnostic tests with the ability to differentially diagnose COVID-19.

Ultra-fast COVID-19 Diagnostic Sensor (UFC-19) is one such alternate sensor based on electrochemical technology which detects the spike protein of SARS-CoV-2 within a second in saliva and water samples [17,18]. Although this sensor was tested for specificity against HIV in the initial stages of pandemic, viruses that closely resemble SARS-CoV-2 were not studied in detail [17]. Several research articles on other COVID-19 diagnostic sensors have studied the specificity against SARS-CoV, MERS-CoV, and Human CoV (H-CoV) since they are closely related to coronaviruses [19,20,21]. Hence, in this paper, ML and DL algorithms were explored to process UFC-19 sensor data. SARS-CoV-2, SARS-CoV, MERS-CoV, H-CoV, and Influenza samples were tested with the UFC-19 and the current response dataset was created. Each sample was diagnosed with a binary classification to predict if they are SARS-CoV-2 positive or negative. To achieve the best accuracy, sensitivity, and specificity, multiple algorithms were compared and the best models were presented to automatically predict the nature of samples tested using the UFC-19.

## 2. Materials and Methods

### 2.1. UFC-19 Sensor

UFC-19 technology is a nickel-based electrochemical sensor that analyzes electrochemical interactions between the sensor probe and SARS-CoV-2 spike proteins contained in saliva or water in the presence of an electrolyte (electrode/electrolyte interface) in less than a second. As described in our previous works, the UFC-19 prototype uses nickel (McMaster-Carr Multipurpose 400 Nickel Rods ¼” diameter, one end machined to 2 mm diameter) as the working electrode, and platinum as the counter and pseudo reference electrodes of size 0.02” in diameter with a length of 2.75” and 1.25” respectively (ESPI metals, Ashland, OR, USA; 3N5 purity) [17,18,22]. The UFC-19 prototypes use commercially available sterile 5 mL screw cap vials (Grainger, Lake Forest, IL, USA, Item# 52JV50) for testing and the sample volume, typically 0.2 mL is diluted in a standardized potassium hydroxide solution (0.1 M from ACROS Organics^TM^, Fair Lawn, NJ, USA, Lot# A0411825) to a total volume 2.0 mL. More details about the method can be found in the literature. The working electrode was rotated during testing at 400 rpm to flow the electrolyte containing SARS-CoV-2 towards the electrode surface [17,18,22]. UFC-19 senses the presence of SARS-CoV-2 electrochemically by producing a current response when an electric potential is applied. When SARS-CoV-2 is present in a sample, a positive current spike compared to a baseline/background (devoid of SARS-CoV-2) current is produced at a short response time ensuring that the SARS-CoV-2 spike protein in the sample has been sensed. It is hypothesized that the positively charged hydrogen occupancies on the SARS-CoV-2 S1 spike protein interacts with the negatively charged electrocatalyst upon the application of voltage resulting in electrostatic charges recorded as current due to electron flow [17].

In this study, SARS-CoV, MERS-CoV, Human CoV (H-CoV), and Influenza were used as confounding organisms since they are closely related with SARS-CoV-2 and influenza is common and highly possible to be present in tested patients. These viruses were assumed to confuse UFC-19 the most and this matrix can further be extended as needed in future studies. The concentration being tested for all the viruses in the matrix was fixed at 0.1 cp/mL. In our previous work (detection of SARS-CoV-2 in air), we had determined that the limit of detection (LoD) was 0.5 cp/mL in aqueous solution collected after the virus was captured in the sample vial [18]. This LoD was related with the air system being tested and includes the viral losses at different components of the air capturing system. However, the LoD of UFC-19 by spiking SARS-CoV-2 directly in de-ionized water was found to be lower. Heat-inactivated SARS-CoV-2 was spiked into de-ionized water directly and diluted serially. According to the metrics set by the Food and Drug Administration for antigen tests, it was determined that 0.1 cp/mL was the LoD since 0.1 cp/mL was the least concentration at which at least 19 out of 20 replicates were reliably detected as positives [23]. Hence, 0.1 cp/mL was chosen for the machine learning dataset collection to assess and incorporate the effect of lowest SARS-CoV-2 signals into the model.

The testing methodology was similar to SARS-CoV-2 when testing other viruses. Sample preparation involved spiking the viruses in de-ionized water following the same procedure that was used for SARS-CoV-2. The only change made was in the solution preparation to adjust the dilutions based on the initial stock concentration of each of the viruses to reach the desired final concentration which is also 0.1 cp/mL. In general, the purchased viral stocks were serially diluted until a concentration 10-times higher than the final testing concentration. To make the samples for testing, 200 µL of the 10-times concentrated solution was taken and diluted to 2 mL using deionized water.

### 2.2. Initial Signature Analysis of SARS-CoV-2 and Comparison with Other Viruses

**Figure 1:**
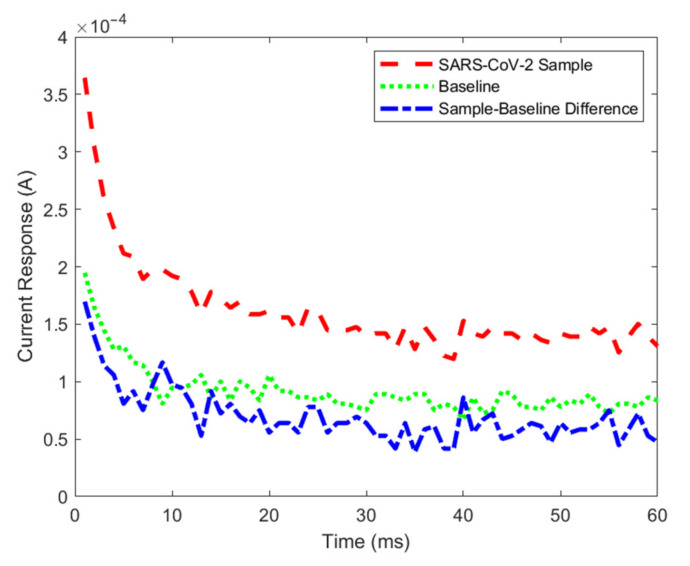
Example current response of a SARS-CoV-2 positive sample with 0.1 cp/mL concentration, baseline (true negative), and sample–baseline difference. The SARS-CoV-2 sample current response is higher than the baseline sample.

To determine the presence or absence of SARS-CoV-2 in samples, chronoamperometry technique was applied using a Gamry Reference 600+ potentiostat (Gamry Instruments, Warminster, PA, USA). The potentiostat applies 0.58 V for 60 ms and the current response is collected with 1 ms intervals and the corresponding 60 ms current response is obtained. First, a baseline sample that is known to be a true negative and then the actual samples are tested. The baseline and sample current responses were compared to determine the result. Based on the interaction between SARS-CoV-2 proteins and the electrodes, a higher current response reading was observed especially at the 1 ms point [17,18,22]. The current response examples of baseline (true negative) and SARS-CoV-2 samples with 0.1 cp/mL concentration are shown in Figure 1. Here, the SARS-CoV-2 sample response (represented in red) is higher than the baseline (represented in green). For simplicity, in the manual data analysis, only 1 ms point was taken into consideration. Thus, in the initial data analysis, it was determined that a sample is declared positive if the sample current at 1 ms is greater than the baseline current by at least 2%. This 2% rule was determined by rigorous testing and analysis of the data to keep the false negative and false positive results at a minimum when the SARS-CoV-2 and blank samples were analyzed. In other words, the following rule was followed in the manual data analysis:

(1) Sample Current (1 ms)−1.02×Baseline Current (1 ms)≤0, sample is negative

(2) Sample Current (1 ms)−1.02×Baseline Current (1 ms)>0, sample is positive

Other viruses such as SARS-CoV, H-CoV, MERS-CoV, and Influenza A were also considered to check if the determined signature rule is valid or not in the presence of other viral particles. The products and vendor information are listed in Table 1. Samples with SARS-CoV-2 (100 samples), SARS-CoV (100 samples), H-CoV (100 samples), MERS-CoV (100 samples), Influenza (100 samples) viruses, and Blank (100 samples) samples were prepared. Since the LoD of SARS-CoV-2 with UFC-19 was determined to be 0.1 cp/mL, the same concentration was considered for all viruses and the same methodology was used to prepare all the virus samples used in this study. All samples were tested with 4 different sensors each operated by a different operator to evaluate variability from the sensor to sensor due to manufacturing and assembly. Each measurement was considered a standalone data point giving rise to 400 data points for each case. Using the data from the 4 sensors and combining them, 6 datasets each consisting of 400 SARS-CoV-2, 400 blank, 400 SARS-CoV, 400 H-CoV, 400 MERS-CoV, and 400 Influenza A signals were obtained as shown in Table 1. Since the goal of this research is to identify the SARS-CoV-2 presence, only SARS-CoV-2 samples were considered as positive, and the rest were considered as negative samples. The results were analyzed with the abovementioned 2% signature rule by just analyzing the current response at 1 ms presented in Section 3.1.

### 2.3. Machine Learning and Deep Learning Algorithms

Although the manual analysis of the data can distinguish the SARS-CoV-2 samples from the blank samples with 100% accuracy, due to similarities between the selected viruses, more advanced data analysis techniques are required. Thus, instead of just analyzing a single point of the dataset at the 1 ms point, the whole 60 ms of the signals were analyzed with ML and DL algorithms to achieve better accuracy, sensitivity, and specificity.

The used dataset for a binary classification between positive and negative samples is shown in Table 2. Since larger datasets always yield higher accuracy in ML and DL applications, 400 more SARS-CoV-2 samples with the same 0.1 cp/mL concentration were added to the dataset as shown in Table 1. Therefore, 800 SARS-CoV-2 samples were considered as positive, and to match this number, 800 negative samples that consist of blank and other virus samples were considered as negatives. Here, the 160 samples were randomly selected from the 400 samples that were previously used in the manual data analysis.

#### 2.3.1. Machine Learning Algorithms with Manual Feature Extraction

Traditional ML algorithms such as AdaBoost Classifier (ABC), Decision Tree Classifier (DTC), Multi-Layer Perceptron Classifier (MLPC), and Support Vector Classifier (SVC) algorithms were considered [29,30,31]. The Scikit-Learn library [32], was used and all data preparation and machine learning were done using python programming language [33]. In these algorithms, manual feature extraction also known as statistical feature engineering is required for the best performance. There are several statistical features that can be derived from the signals. While this can be a cumbersome task, the most popular ones such as maximum, minimum, average, standard deviation, etc., values should always be considered [34]. In this study, 17 different features are used which are listed in Table 3 with their corresponding equations. While the F0 feature is the feature used in the manual signature analysis, the rest of the features are derived from the current response difference between sample and baseline readings.

**Figure 2:**
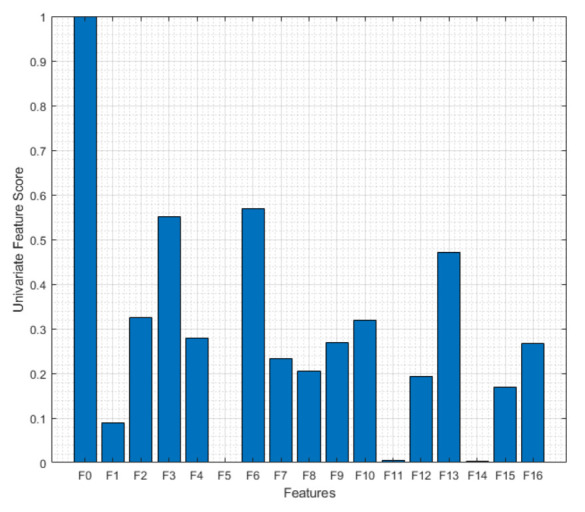
Univariate feature scores of the futures. The bar chart shows the informative and non-informative features. The most informative features are F0, F6, and F3 and the least informative features are F5, F11, and F14.

Although it is easy to tell F0 is a significant feature in this study, it is hard to tell what other features carry important information about the signals. When non-important features are also fed to ML algorithms, it can cause harm to the performance. That is why it is also important to eliminate non-informative features. Figure 2 shows the univariate feature scores of each feature which are calculated by assessing if there is any significant relationship between the features and their labels with an ANOVA F-test [35]. According to this chart, the three most informative features are F0, F6, and F3 and the least informative features are F5, F11, and F14. As aforementioned, F0 was expected to be an important feature, here it was seen that mean value (F3) and trimmed mean value (F6) also play an important role while harmonic mean (F5), crest factor (F11), and Kurtosis (F14) do not carry significant important information.

It is important to study which combination of features can provide the best results. To examine that, the elimination of less informative features was conducted. First, ML algorithms were fed with all features, of which five features with the lowest scores were eliminated. Consecutively, more features were eliminated as shown in Table 4 until there was only the F0 feature left, which is the most informative feature of all. This way, the classification performance of the ML algorithms with different sets of features were compared to select the best algorithm with the most informative combination of the features.

For all ML algorithms, 80% of the data (1280 samples) were randomly selected and used for training and 20% of the data (320 samples) were used for testing. Each algorithm has several hyperparameters that need tuning for the best results. This was done by using a grid search algorithm. The grid search algorithm explores the results of algorithms with a wide range of hyper-parameter variables and compares the results of each combination. Hyper-parameters of each algorithm were determined for the best accuracy, sensitivity, and specificity and used in further testing. To check the consistency of the results, all algorithms were run 25 times repeatedly with the randomly selected training and testing samples. This method would also check the robustness of the algorithms to see if they are sensitive to the training sample subset.

#### 2.3.2. Convolution Neural Networks (CNN)

The CNN is a deep learning algorithm that can process images or time series of data without the need for preprocessing or any other feature extraction method. It has been widely used in SARS-CoV-2 diagnostic studies with high sensitivity and specificity [7,8]. While most of these studies have used X-ray images and reverse transcription polymerase chain reaction (RT-PCR) datasets, in this study, the current response from chronoamperometry application was used as input. To build the CNN algorithm, a sequential model using the Keras library was used. Initially, a standard network that consists of a single convolutional layer followed by fully connected layers was determined as a starting point and tuned for best performance. The convolutional layers scan the data with the determined window (kernel size) and extract important data by filtering. The fully connected layers use the extracted features and learn the non-linear relation between the data and the labels. Dropout layers were also utilized after each convolutional and fully connected layers to prevent overfitting. By increasing or decreasing the number of layers and changing the parameters of each layer, better results can be obtained. Thus, extensive tuning of the network parameters was exercised to obtain the best-performing algorithm. Different combinations of the number of layers, activation functions, kernel sizes, number of filters, and optimizers were run, and the network parameters were finalized according to the best accuracy, sensitivity, and specificity results.

As aforementioned, the current response collected from the samples consists of 60 ms data that is collected with 1 ms intervals. In CNN, the difference between the sample and the baseline readings was used which consists of 60 points of time series of data. As it is shown in Table 2, 800 signals of SARS-CoV-2 signals were labeled as positive, and 800 signals which consist of other viruses and blank samples were labeled as negative and used in the CNN algorithm. The total dataset was randomly divided into 60%, 20%, and 20% for CNN training, validation, and testing purposes. While labeled training and validation datasets were used for training and training validation, the remaining 20% of test samples were used to check the performance of the trained algorithm. Here, it was also important to analyze if all 60 ms of the data carry important information or not. Thus, different portions of these 60 ms data (0–1, 0–5, 0–10, 0–20, 0–40, 0–50, and 0–60 ms) were used as the input of the CNN and impact on the diagnostic performance analyzed. The overall accuracies and confusion matrices are presented for each case in Section 3.2.2.

## 3. Results and Discussion

### 3.1. Results of Initial Signature Analysis of SARS-CoV-2 and Comparison with Other Viruses

**Figure 3:**
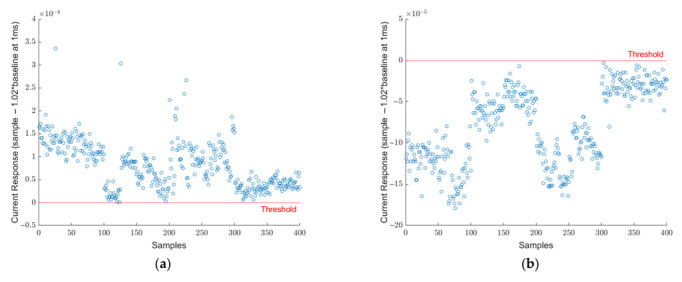
(**a**) Signature analysis of 400 SARS-CoV-2 positive samples. All current response difference points for 400 samples are above the threshold line. Meaning all samples were diagnosed correctly as SARS-CoV-2 positive. (**b**) Signature analysis of 400 blank samples. All current response difference points for 400 samples are below the threshold line. Meaning all samples were diagnosed correctly as SARS-CoV-2 negative.

**Figure 4:**
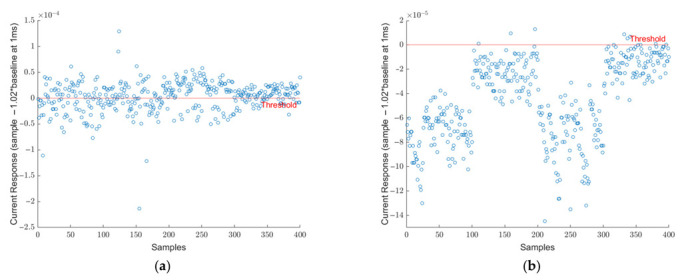
(**a**) Signature analysis of 400 SARS-CoV samples. The 242 out of 400 samples were misclassified as SARS-CoV-2 positive and the rest of the samples falls below the threshold line (**b**) Signature analysis of 400 HCoV-OC43 samples. Most of the samples were correctly classified as SARS-CoV-2 negative. Only 9 samples were classified as SARS-CoV-2 positive.

The collected dataset that was presented in Table 1 was investigated by analyzing the 1 ms data point as mentioned in equations 1 and 2. Each sample of the SARS-CoV-2 virus is plotted in Figure 3a. In this figure, each data point represents the difference between the current response of SARS-CoV-2 and 102% of the baseline at the 1 ms point. The red line at y = 0 represents the threshold limit for the decision point. If the difference falls below the red line, the sample is called negative and positive otherwise. It can be seen that all 400 points fall above the threshold limit and, therefore, they are all correctly identified as SARS-CoV-2 true positive samples. Similarly, in Figure 3b, the 400 blank samples are demonstrated. Here, all samples fall below the 0-line meaning they are all identified correctly as true negatives. As aforementioned, this threshold was determined specifically to distinguish SARS-CoV-2 positive samples from blank samples. As research progressed, different viruses were included in the scope. Figure 4a shows the signature analysis of 400 SARS-CoV samples. Out of 400 samples, 242 were misclassified as SARS-CoV-2 positive, and the rest of the samples falls below the threshold line which are considered SARS-CoV-2 negative. Figure 4b shows the results for 400 HCoV samples. Most of the HCoV samples were correctly classified as SARS-CoV-2 negative. Only 9 samples were classified as SARS-CoV-2 positive. This shows that HCoV is easy to distinguish from SARS-CoV-2 samples, unlike SARS-CoV.

**Figure 5:**
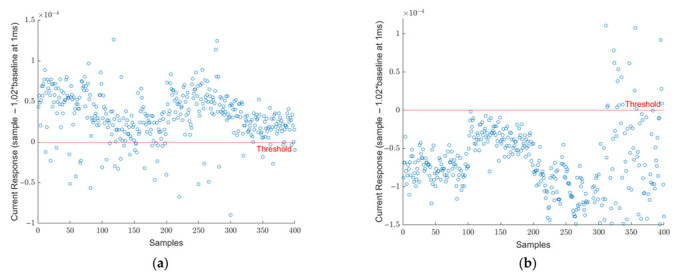
(**a**) Signature analysis of 400 MERS-CoV samples, while 356 samples were above the threshold and classified as SARS-CoV-2, 44 samples were under the threshold. (**b**) Signature analysis of 400 Influenza virus samples, only 14 samples are identified as SARS-CoV-2 positive.

The last two virus sample results are presented in Figure 5a,b for MERS-CoV and Influenza. Figure 5a shows the results for 400 MERS-CoV virus readings, here we observed that the majority of the samples (356 out of 400) were identified as SARS-CoV-2 positive. Finally, Figure 5b shows the results for 400 Influenza virus readings. It can be seen from this figure that the majority of Influenza virus samples were successfully identified as SARS-CoV-2 negative. Only 14 samples are identified as SARS-CoV-2 positive.

**Figure 6:**
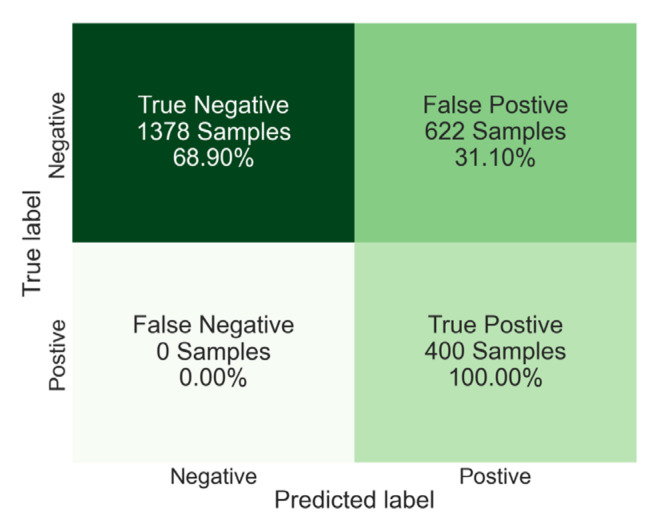
Confusion matrix of manual data analysis results with 2% difference threshold rule. The true-positive rate is 100% since the threshold is set for a 100% sensitivity rate. However, due to the high false-positive rate, the overall accuracy is 74.1%, precision is 39.1% and F1 score is 56.3%.

These results show that HCoV and Influenza are easier to distinguish from SARS-CoV-2. But SARS-CoV and MERS-CoV viruses show very close signatures to SARS-CoV-2. A reason for this could be due to the high similarity in their viral morphology and spike protein characteristics with SARS-CoV-2 [36]. This is causing a very high rate of SARS-CoV-2 false-positive results. Although these viruses were almost eradicated and very rare [37,38], it can be beneficial to be able to create a study that can distinguish coronaviruses from each other. Figure 6 shows the confusion matrix results of all manual analyses of the samples. Since the manual analysis 2% rule is specifically designed for high sensitivity. A 100% true positive rate (TPR) is achieved. However, due to the high misclassification of MERS-CoV and SARS-CoV virus samples as false-positive samples (31.1%), the specificity, which is also known as true negative rate (TNR), is calculated to be 68.9% with a dissatisfactory overall accuracy of 74.1%.

### 3.2. Machine Learning and Deep Learning Results

#### 3.2.1. Machine Learning

**Figure 7:**
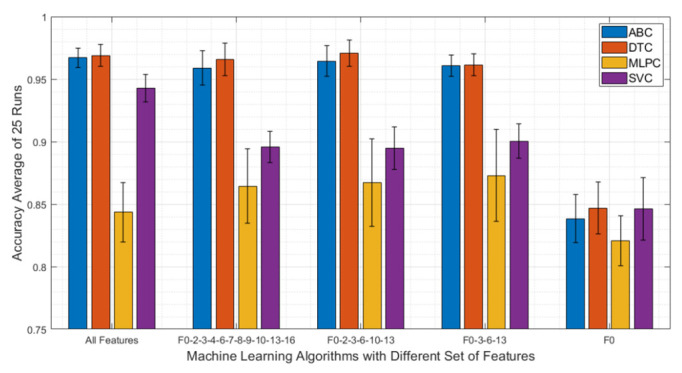
Machine learning algorithm result comparison for different sets of features of ABC, DTC, MLPC, and SVC algorithms. The DTC algorithm outperformed all other algorithms with the feature set of F0-F2-F3-F6-F10-F13 by achieving 96.6% overall accuracy.

After all four machine learning algorithms were tuned for their best performances, different feature combinations that are listed in Table 2 were fed to all algorithms and run 25 times. Figure 7 shows the accuracy and standard deviation results for a different set of features. All algorithms performed the worst when F0 is used as the only feature. On the other hand, when more features are included the performance of the algorithms vary. For instance, SVC achieved its best accuracy (89.1%) when all features were included. On the other hand, ABC and DTC yielded the highest accuracies (96.3% and 96.6%, respectively) when only F0, F2, F3, F10, and F13 were used. This shows that the performances of the algorithms depend on the selected feature set and extensive feature engineering should be conducted.

**Figure 8:**
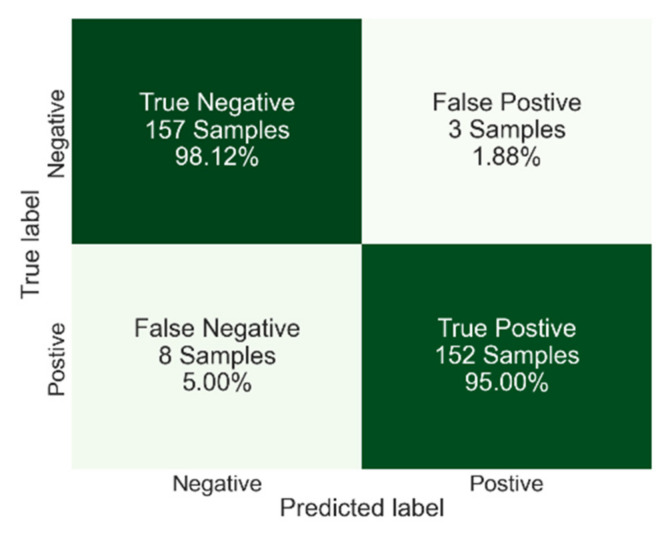
Confusion matrix results for DTC algorithm with an overall accuracy of 96.6%.

The highest average accuracy overall was achieved by DTC with 96.6% accuracy and the lowest standard deviation of 0.01. Figure 8 shows the confusion matrix results of DTC with the feature set of F0-F2-F3-F6-F10-F13. This figure provides more insight into the classification results on top of the accuracy. Here we can see that only three samples were classified as false positive, and eight samples were classified as false negative. This yields 95.00% sensitivity and 98.12% specificity.

#### 3.2.2. Deep Learning Results

**Figure 9:**
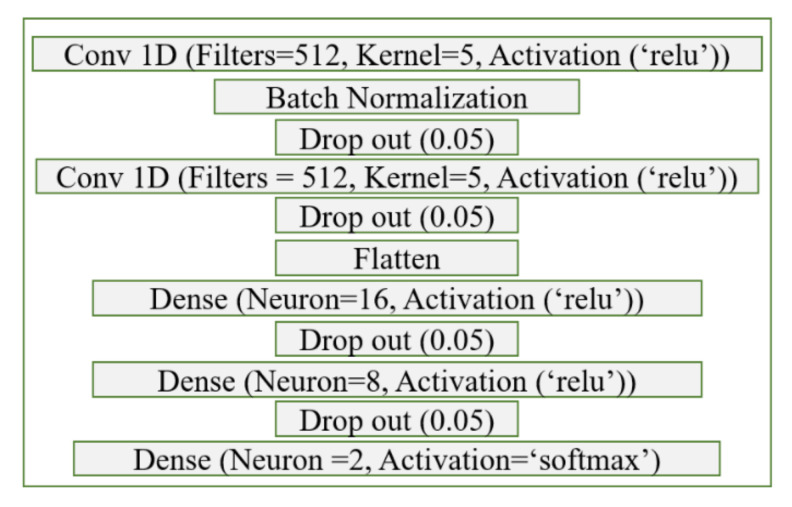
The CNN network parameters are tuned for optimal performance.

The model parameters of CNN were determined for the best classification results by trying different layer numbers, activation functions, filter sizes, neuron numbers, etc. The finalized model aims to achieve the highest repeatable accuracy, sensitivity, and specificity with the different randomly selected subsets of training data. This finalized network consists of two convolutional layers connected to three dense layers where the first four layers are activated with the relu function, and the last layer is activated with the softmax function. After each convolution layer and dense layer, a dropout layer with 0.10 rate was implemented to minimize the overfitting. The summary of the CNN algorithm used is shown in Figure 9.

**Figure 10:**
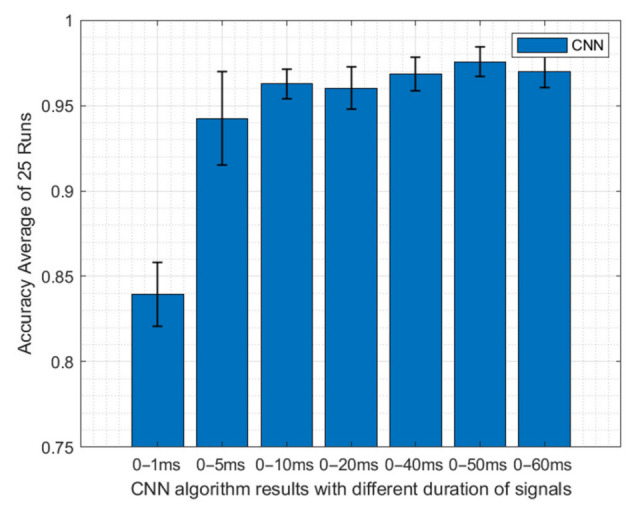
Accuracy and standard deviation results of CNN algorithm with the different time window of the data. The results showed that the highest accuracy with the lowest variation was achieved by using the 0–50 ms portion of the signals.

Since it is hard to tell if all 60 ms of the data carry important information, different portions of the 60 ms were fed to this algorithm to optimize the performance of the CNN algorithm. Thus, 0–1, 0–5, 0–10, 0–20, 0–40, 0–50, and 0–60 ms portions of the dataset were fed to the CNN, and training and testing were done 25 times with randomly selected training, validation, and testing datasets. Figure 10 shows the accuracy and standard deviation results for different windows of the data. While this figure shows the lowest accuracy result was obtained using only 0–1 ms of the data, as the signal window gets larger the average accuracy of 25 runs increases and variation decreases except for the 0–60 ms window. It is interesting to note that the peak accuracy was achieved with the signal that contains the 0–50 ms portion of the signal. When the last 10 ms of the data was also included a slight decrease in accuracy was observed. The chronoamperometry signals capture the virus–electrocatalyst interaction at the double layer. At longer times, the SARS-CoV-2 concentration in bulk is captured rather than the concentration at the double layer. This could possibly explain the drop in accuracy when the last 10 ms of the current response is fed to the CNN algorithm. In other words, the 50–60 ms portion of the signal consists of more noise than the signal and causes confusion when included in the training dataset. Thus, only the 0–50 ms window of the signals was considered in the CNN algorithm classification training and testing for SARS-CoV-2 detection.

**Figure 11:**
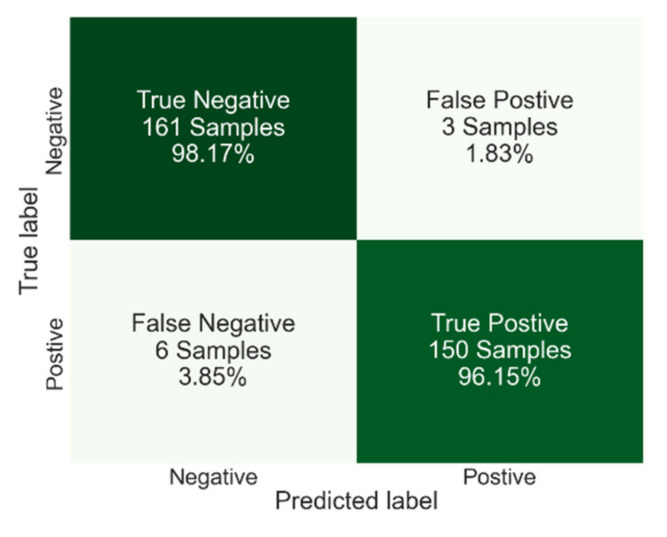
Confusion matrix results for the CNN algorithm with an overall accuracy of 97.20%, specificity of 98.17%, and sensitivity of 96.15%.

Figure 11 shows the confusion matrix for an average of 25 CNN algorithm runs when the 0–50 ms portion of the signals was used as the input. The overall accuracy achieved here is 97.2% with 0.0085 standard deviation which are slightly better than DTC results. When the results of CNN are further compared to DTC, it can be seen that they have identical FPR (1.83%) however, CNN has a slightly lower FNR. In other words, while the specificity of CNN and DTC (98.17% and 98.12, respectively) are very close to each other, the sensitivity of CNN (96.15%) is higher compared to the sensitivity of the DTC (95%).

**Figure 12:**
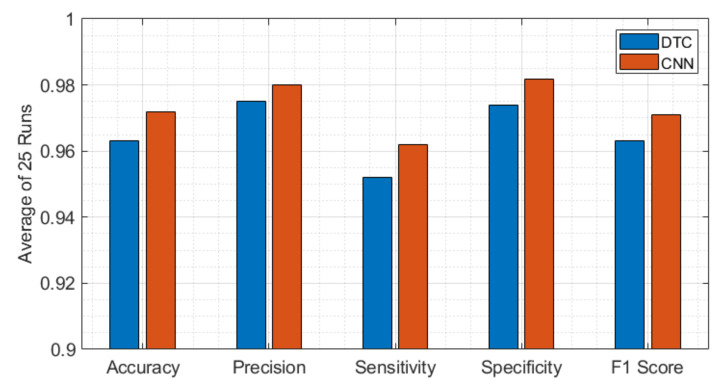
Performance metric comparison between DTC and CNN to diagnose SARS-CoV-2 with their best performing parameters. While the CNN algorithm outperforms the DTC algorithm in accuracy, precision, sensitivity, specificity, and F1 score.

Figure 12 compares the other classification performance matrixes of DTC and CNN side by side. This figure illustrates that CNN outperforms the DTC algorithm in accuracy, precision, sensitivity, specificity, and F1 score.

## 4. Conclusions

This study demonstrates detection of SARS-CoV-2 selectively in the presence of other viruses such as SARS-CoV, HCoV-OC43, MERS-CoV, and Influenza A using an electrochemical sensor (UFC-19) and analyzing the data using ML and DL algorithms. A dataset was created by testing 800 SARS-CoV-2 samples, 160 samples for every other virus, and blank samples. Various machine learning and deep learning algorithms were employed to analyze the data and aim for the highest detection accuracy, sensitivity, and specificity. While the different sets of features were explored for the machine learning algorithms, different windows of the signals were considered for the deep learning algorithm. All algorithms were fine-tuned to achieve the highest possible performance and run 25 times with the randomly selected training and testing datasets to cross-validate the algorithms with different subsets of data. The cross-validated results of each algorithm were compared, and it has been shown that the CNN algorithm outperforms ABC, DTC, MLPC, and SVC algorithms in every diagnostic metric. This study demonstrates that the UFC-19 sensor with a combination of a DL algorithm can detect the SARS-CoV-2 virus against SARS-CoV, H-CoV, MERS-CoV, and Influenza A with an accuracy of 97.20%, a specificity of 98.17%, and a sensitivity of 96.15%. These specificity and sensitivity results are comparable with other deep learning studies that only analyze SARS-CoV-2, or SARS-CoV-2 and influenza viruses with more expensive and time-consuming RT-PCR or X-ray imaging data [7,8,39]. This study is important to show that an electrochemical sensor can distinguish SARS-CoV-2 from the other coronaviruses and influenza virus in a very fast manner with high sensitivity and specificity. Although we believe that we can detect the other viruses in saliva, only SARS-CoV-2 detection in saliva has been demonstrated [17]. Successful detection of other viruses in saliva would expand the scope of this sensor for the clinical detection of other viral diseases, apart from being used for surveillance in air. It is recommended that future studies focus on selectively identifying H-CoV, Influenza, and SARS-CoV-2 viruses as well as co-infections. This would help to inform the tested patient to know what they are infected with and prevent further spread of infection.

## Figures and Tables

**Table 1:** The dataset used for initial manual data analysis with 2% signature rule.

Samples	Vendor Product	Number of Samples	Label
SARS-CoV-2	ATCC VR-1986HK [24]	400	Positive
Blank	NA	400	Negative
SARS-CoV	ZeptoMetrix NATSARS-ST [25]	400	Negative
H-CoV OC43	ZeptoMetrix 0810024CFHI [26]	400	Negative
MERS-CoV	ZeptoMetrix NATMERS-ST [27]	400	Negative
H1N1 Influenza A	ZeptoMetrix 0810109CFNHI [28]	400	Negative

**Table 2:** The dataset used in machine learning to diagnose SARS-CoV-2.

Sample	Number of Samples	Label
SARS-CoV-2	800	Positive
SARS-CoV	160	Negative
Influenza	160	Negative
H-CoV	160	Negative
MERS-COV	160	Negative
Blank	160	Negative

**Table 3:** List of statistical features extracted from sensor reading that was used to train test machine learning algorithms (ABC, DTC, MLPC, and SVC).

#	Name	Definition	#	Name	Definition
F0	2% current difference	Samp(1 ms)−1.02Base(1 ms)	F9	Mean absolute deviation	$\frac{1}{N}\overset{N}{\underset{1}{\sum }}\left|x\left(n\right)-\bar{x}\right|$
F1	Maximum value	maxx(n)	F10	Median absolute deviation	$\frac{1}{N}\overset{N}{\underset{1}{\sum }}\left|x\left(n\right)-x_{median}\right|$
F2	Minimum value	minx(n)	F11	Crest Factor	$\frac{max\left(x\left(n\right)\right)}{\sqrt{\frac{1}{N}\sum_{1}^{N}{\left(x\left(n\right)\right)}^{2}}}$
F3	Mean	$\frac{1}{N}\overset{N}{\underset{1}{\sum }}x\left(n\right)$	F12	Peak2RMS	$\frac{max\left(\left|x\left(n\right)\right|\right)}{\sqrt{\frac{1}{N}\sum_{1}^{N}{\left(x\left(n\right)\right)}^{2}}}$
F4	Peak to peak	F0−F1	F13	Skewness	$\frac{\frac{1}{N}\sum_{1}^{N}{\left(x\left(n\right)-\bar{x}\right)}^{3}}{\frac{1}{N}\sum_{1}^{N}{\left(x\left(n\right)-\bar{x}\right)}^{2}}$
F5	Harmonic mean	$\frac{N}{\sum_{1}^{N}1/x\left(n\right)}$	F14	Kurtosis	$\frac{\frac{1}{N}\sum_{1}^{N}{\left(x\left(n\right)-\bar{x}\right)}^{4}}{{\left(\frac{1}{N}\sum_{1}^{N}{\left(x\left(n\right)-\bar{x}\right)}^{2}\right)}^{2}}$
F6	Trimmed mean	Mean excluding outliers	F15	Shape Factor	$\frac{\sqrt{\frac{1}{N}\sum_{1}^{N}{\left(x\left(n\right)\right)}^{2}}}{\frac{1}{N}\sum_{1}^{N}\left|x\left(n\right)\right|}$
F7	Variance	$\frac{1}{N}\overset{N}{\underset{1}{\sum }}{\left(x\left(n\right)-\bar{x}\right)}^{2}$	F16	RMS	$\sqrt{\frac{1}{N}\overset{N}{\underset{1}{\sum }}{\left(x\left(n\right)\right)}^{2}}$
F8	Standard deviation	$\sqrt{\frac{1}{N}\overset{N}{\underset{1}{\sum }}{\left(x\left(n\right)-\bar{x}\right)}^{2}}$	Where x(n)=Sample(n)−baseline(n)		

**Table 4:** The list of feature sets that were used in ML algorithms. It was started with all features and the elimination of less informative features was applied.

Eliminated Features	Feature Numbers
None Eliminated	F0 F1 F2 F3 F4 F5 F6 F7 F8 F9 F10 F11 F12 F13 F14 F15 F16
Features with 5 Lowest Scores	F0 F2 F3 F4 F6 F7 F8 F9 F10 F13 F16
Features with 10 Lowest Scores	F0 F2 F3 F6 F10 F13
Features with 12 Lowest Scores	F0 F3 F6 F13
Features with 16 Lowest Scores	F0

## Data Availability

The data generated and used for this study are available upon request.
